# Development and clinical usability of a new traction device “medical ring” for endoscopic submucosal dissection of early gastric cancer

**DOI:** 10.1007/s00464-013-2887-6

**Published:** 2013-03-23

**Authors:** Kenshi Matsumoto, Akihito Nagahara, Hiroya Ueyama, Hironori Konuma, Takasi Morimoto, Hitoshi Sasaki, Takuo Hayashi, Tomoyoshi Shibuya, Naoto Sakamoto, Taro Osada, Tatsuo Ogihara, Takashi Yao, Sumio Watanabe

**Affiliations:** 1 Department of Gastroenterology, Juntendo University School of Medicine, 2-1-1 Hongo, Bunkyo-ku, Tokyo, 113-8421 Japan; 2 Department of Human Pathology, Juntendo University School of Medicine, 2-1-1 Hongo, Bunkyo-ku, Tokyo, 113-8421 Japan

**Keywords:** Endoscopic submucosal dissection, Traction device, Can approach anal side and oral side, No harmful effect on the body, Easy use and cost effective

## Abstract

**Background:**

Although various traction devices exist for endoscopic submucosal dissection (ESD), the effects of the material used in the devices on the human body has not been considered. Moreover, there has been no report on a device that facilitates dissection both on the oral and anal side of the lesion. We made a traction device that has no deleterious effects on the body and is noninvasive, easy to use, and enables a bilateral approach in ESD. We report the process of its creation and a prospective evaluation of its usage in actual ESD procedures.

**Methods:**

This study is prospective case control study. Thirty-seven patients for whom the device would be used were consecutively and prospectively enrolled (device used group). Control subjects in whom the device would not be used and who had lesions matched for size and location with those of the device used group were randomly selected (device not used group). Both groups were classified into three subgroups according to treatment difficulty: group A: easy; group B: intermediate; and group C: difficult. The dissection time per cm^2^ in each group was examined.

**Results:**

Dissection times in the device not used group/device used group were as follows: group A, 5.8/2.1 min/cm^2^ (*p* < 0.01); group B, 6.1/3.8 min/cm^2^ (*p* < 0.05); and group C, 7.9/3.6 min/cm^2^ (*p* < 0.01), respectively.

**Conclusions:**

The newly developed medical ring was shown to be feasible and safe and allowed excellent visualization through suitable tension and facilitated rapid gastric ESD.

**Electronic supplementary material:**

The online version of this article (doi:10.1007/s00464-013-2887-6) contains supplementary material, which is available to authorized users.

It has been reported that endoscopic submucosal dissection (ESD) of early gastric cancer (EGC) improves the rate of successful en bloc resection [1, 2]. ESD has been rapidly gaining popularity worldwide, primarily because of its ability to remove larger EGCs en bloc, thus reducing local recurrence caused by piecemeal resection [3]. However, ESD is associated with more complications, such as bleeding and perforation, and certainly requires more skillful endoscopic techniques [4–6] and a longer procedure time than conventional endoscopic mucosal resection (EMR). Major factors that make ESD difficult are risks of bleeding and perforation due to the blind approach to the cut line of the submucosal layer. The cut edge of the lesion curls inward and obscures the endoscopist’s view. Moreover, because of the necessity of getting into the lesion to have visual contact with the submucosal layer until the mucosal lesion is separated, the distance of the scope is extremely close and visualization of the field is narrow, so the cleaving direction is unclear.

Endoscopic resection should be safe, effective, and applicable to a wide variety of clinical situations. When traction is applied, not only should the submucosal layer be visually recognized, but the scope should be at a suitable distance so that the cleaving direction also is intelligible. Various methods used to pull up the submucosal layer to facilitate visibility have been reported [7–12]. However, a device for this purpose that is not invasive and is convenient, cost effective, sanitary, and safe has not been developed. For traction devices employed within the stomach, no report has mentioned their effect on the body. Also, when such a traction device is detached within the stomach, it passes into the intestine to be eliminated. Furthermore, most devices lift only one part of a lesion, which means that resection can only be performed from one side. Thus, the use of some devices is limited depending on the tumor’s location [9–13]. To facilitate a complicated standard ESD procedure performed using a single working-channel gastroscope (one-hand surgery), we therefore designed a new traction device, the “medical ring,” which is comprised of material already employed in medical devices, has no harmful effect on the body, is easy-to-use, requires no special clip and sheath, and can approach the lesion bilaterally (Okamoto Co., Ltd., Tokyo, Japan; Fig. 1). We previously reported use of this device as a short form technical notes [14], and we reported its usage, although differing from our procedure, for colorectal tumor [15, 16]. However, we were not reported the process of its creation and a prospective evaluation of its usage in actual ESD procedures. We evaluated these issues in this study.

**Fig. 1:**
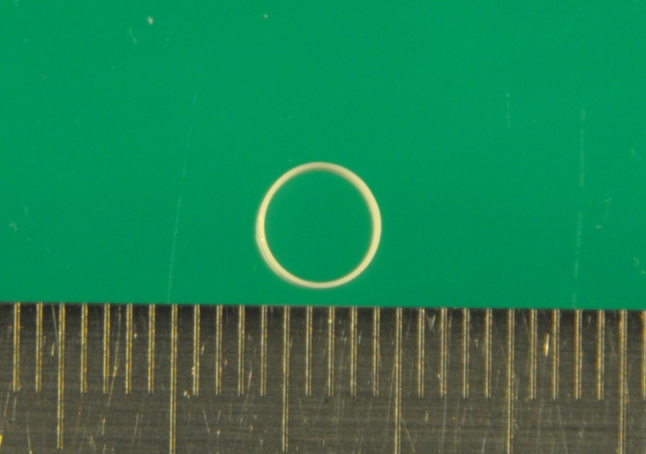
Complete medical ring

## Patients and methods

### Development of the medical ring

The medical ring comprises an inert elastic band, which is made of the same material as used in several medical devices, including the endoscopic variceal ligation (EVL) O-ring, sterile gloves for surgical operations, etc. This elastic band does not dissolve in the gut nor does it deteriorate when sterilized. Moreover, this material successfully underwent examinations for intracutaneous reactions, allergic reactions of the skin, systemic reactions to implantation of the material, and hemolytic reactions in animal models. In consideration of intragastric visibility, the device, which can be stored in a sheath, opens when it gets wet. The sheath can be passed through the instrument channel of a standard endoscope and operability is good. To be consistent with the above considerations, a study was conducted in a porcine model regarding color, diameter, width, thickness, maximum elongation rate against loads, and length of the hemoclip with best usability. Finally, using the completed device in the stomach of a pig, we determined the maximum size lesion for which the device can be used in endoscopy.

### Clinical study of ESD

Between April 2009 and August 2010, patients with gastric adenomas or EGCs more than 10 mm in diameter for whom this device would be used were consecutively and prospectively enrolled (device used group). Control subjects in whom the device would not be used and who had lesions matched for size and location with those of the study group were randomly selected (device not used group).

All lesions were confirmed by histologic evaluation of forceps biopsy specimens before the ESD procedure. As previously reported [17, 18], indications for ESD were restricted to gastric adenoma and differentiated gastric adenocarcinoma. Both the device used and device not used groups were classified into three subgroups according to treatment difficulty: group A, easy (lesion on anterior wall, posterior wall and greater curvature of antrum); group B, intermediate (frontal view of the antrum, lesion over an angle or on the lesser curvature of corpus); and group C, difficult (lesion on the greater curvature of corpus, fornix). After the circumferential cut, the dissection time per cm^2^ in each group and occurrence of complications were examined. Treatment was performed by five operators at an elementary to intermediate level with experience of 30 or fewer cases.

Patients with a latex allergy, advanced malignancy in other organs, fibrosis, submucosal deep invasion, or diffuse-type histology were excluded from this study. The ethics committee approved the study, and detailed written, informed consent was obtained from each patient. The present study was conducted according to the declaration of Helsinki.

### Medical ring-assisted ESD

**Fig. 2:**
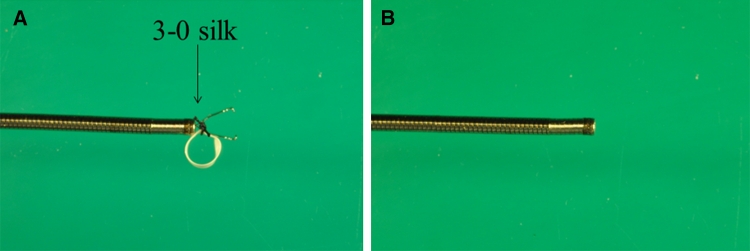
**A** At the time of mounting. Mounting only requires connecting the 3–0 silk to the hemoclip. **B** At the time of storage, device is completely stored in a sheath

All patients were sedated by intravenous injection of midazolam (3–5 mg) and opistan (35 mg). If the patient began to awaken, 2 mg of midazolam was added at the time of each awakening. ESD procedure was initially started using a standard gastroscope with a single working channel (GIF Q260 or Q260 J; Olympus Optical Co., Ltd., Tokyo, Japan). A short, disposable transparent hood (D-201-10704, D-201-11804; Olympus) was attached to the endoscopic tip to make the lesion more visible. A flexible overtube (Sumitomo Bakelite Co., Ltd., Tokyo, Japan) was inserted, which enabled the endoscope to be inserted and retrieved repeatedly and also assisted in aspiration prevention. Marking dots were placed approximately 5 mm outside the margin of the lesions by using APC, forced APC current 1.6L, 50 W (ICC-APC300; ERBE). First, diluted epinephrine (1:100,000) and indigocarmine (1.00 %) was injected to raise the submucosal layer and to insert the tip of the IT-knife2 (KD-611L; Olympus) into the submucosal layer. Then, a small initial incision was made by a flex-knife (KD-630L; Olympus) by using an 80 W, effect 3 Endocut (ICC350; ERBE). Mucosal cutting at the periphery of the marking dots was circumferentially performed with an IT-knife2 with an 80 W Endocut. According to the standard ESD, after circumferential mucosal cutting by an IT-knife2, the procedure was switched to a medical ring-assisted ESD. For mounting, the medical ring is attached to the hemoclip, which was not fully open to avoid closure (HX-610-090; Olympus). The medical ring is attached to the hemoclip with 3–0 silk so that it can be retracted into and stored within the sheath (Fig. 2A). Thus, the sheath contains the device with the hemoclip (Fig. 2B; Video 1). The ESD procedure using the device was performed as follows.

**Fig. 3:**
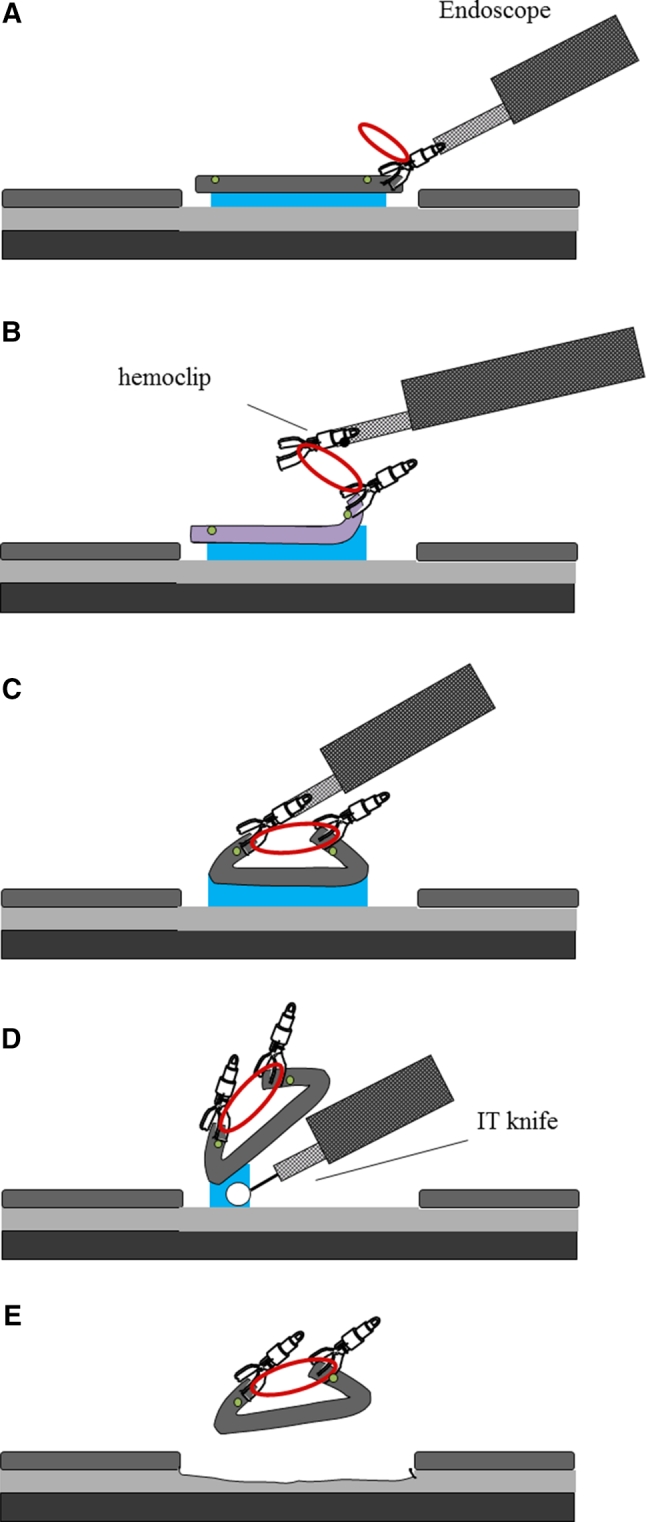
**A** *Marking dots* are made on the circumference of the target tumor, outlining the margin of the lesion. After injection of a saline solution into the submucosa, the tumor is separated from the surrounding normal mucosa by complete incision around the lesion using the IT knife. **B**, **C** The device connects to the edge of the exfoliated mucosa and the opposite side of the lesion. **D** In pulling the lesion up and opening the resection margin, dissection can be rapidly accomplished by tension from the elastic material. **E** After dissection, the device is recoverable with the lesion. Device can be easily removed from the lesion with forceps

**Fig. 4:**
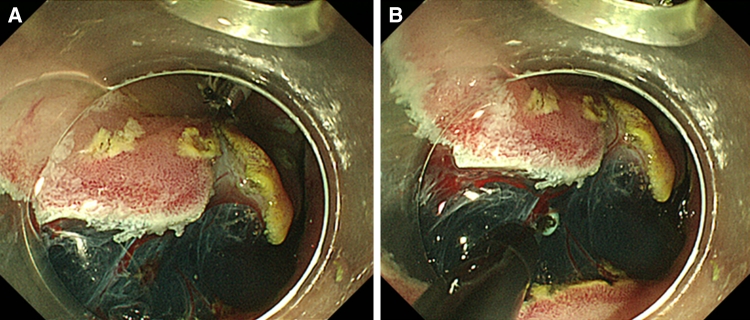
**A** At the time of completion of attachment of the device, the SM layer and blood vessels are visible. **B** Knife contacts can be checked clearly

**Fig. 5:**
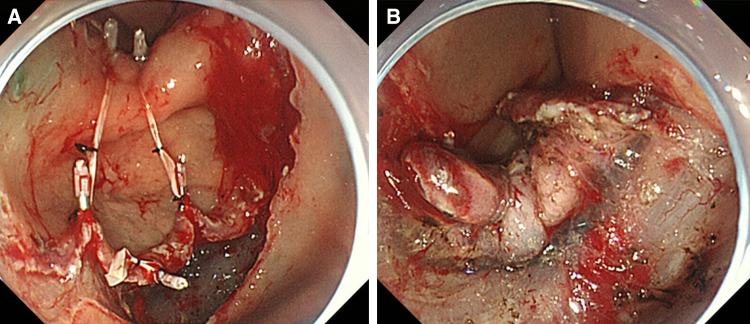
Multiple connections to a large lesion. Multiple connections by the medical ring for resection of a large lesion. If connected with 3–0 silk, two pieces can be stored in a sheath, and it also will be possible to connect them with clips on the* outside *of the sheath. In that case, there will be no limit to the number

First, a tube catheter with a mounted device was passed through the working channel of the gastroscope. Second, the device was connected to the edge of the exfoliated mucosa and the opposite side of the lesion (Fig. 3A–C). There is no need to withdraw the endoscope during the procedure described. The submucosal dissection by an IT-knife2 was performed by suitable tissue tension with hands-free stabilization and visualization (Figs. 3D,  4A, B; Videos 2 and 3). In the case of a large lesion, when dissection cannot be accomplished with one device that could span both sides of the lesion, additional devices may be used (Fig. 5A, B; Video 4). After endoscopic resection, both the resected tissue and device were retrieved into the overtube by a grasping forceps and removed from the stomach (Fig. 3E). Thus, the device can be easily removed with forceps together with the lesion.

### Statistics

Based on data from a preliminary study, the required sample size in this study was 22 subjects per group for a significance level of 0.05 (two-sided, *α* = 0.05, *β* = 0.1). In consideration of the possibility of subjects dropping out, we calculated that a minimum of 30 subjects per group would be required for this study. Data were analyzed using Fisher’s exact test or the *χ* ^2^ test. Odds ratios, absolute differences, 95 % confidence intervals (95 % CI), and *p* values are reported. Statistical significance was defined as *p* < 0.05. All statistical analyses were performed using SAS_ version 8.2 (SAS Institute, Cary, NC).

## Results

### Optimal configuration of the medical ring

**Fig. 6:**
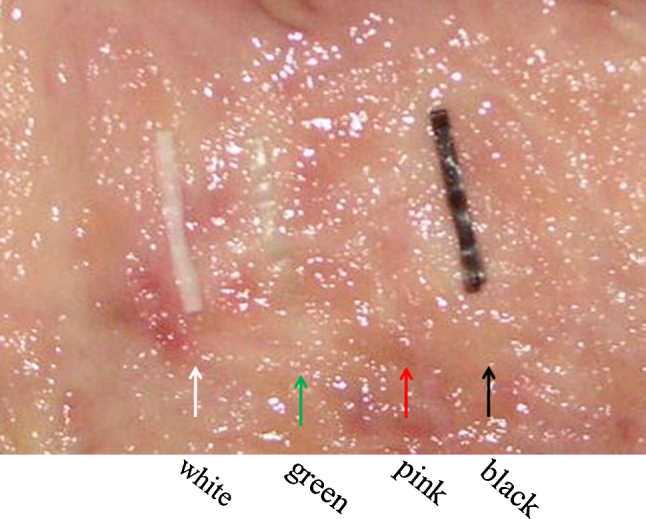
*Pink* and *green* had poor visibility. Although *whites* and *blacks* were excellent in visibility, since silk was *black*, *white* was selected

**Fig. 7:**
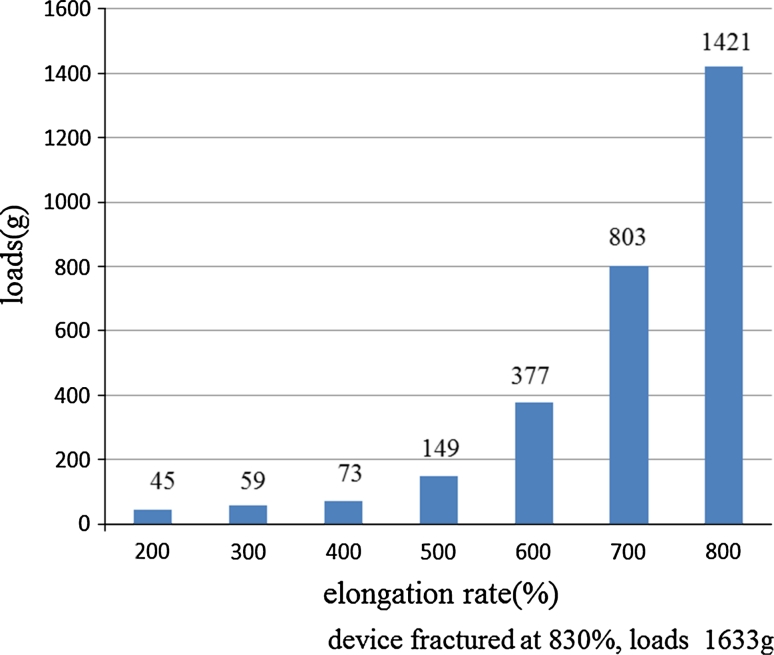
Maximum elongation rate against loads

**Fig. 8:**
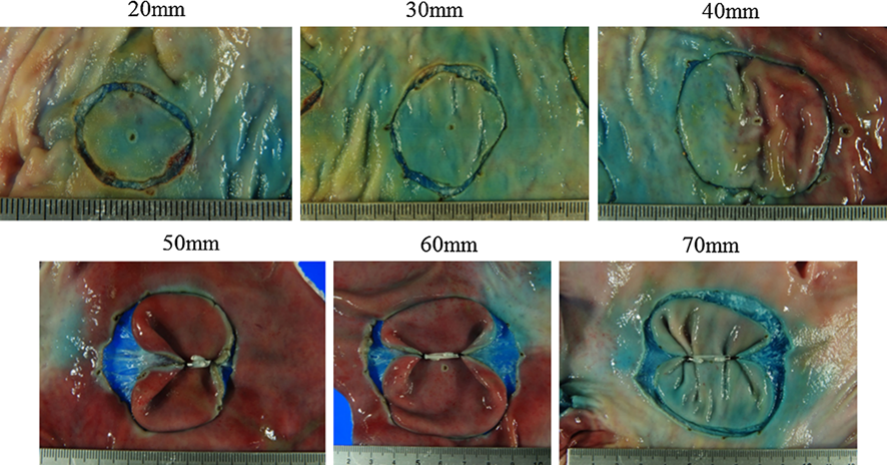
The examination of sizes of lesions in a pig’s stomach that can be dissected by one device under endoscopic control

As to color, we found that pink and green were assimilated to the color of background mucosa; both had poor visibility (Fig. 6). Although both white and black due to contrast had excellent visibility, because the medical ring was connected to the hemoclip by black 3–0 silk, white was selected. Narrower or thicker than 3–0, it is not fit to be connected. Moreover, nylon is not suitable, because it often becomes loose after it is connected. Considering connection to the clip, storability in the sheath and the best ESD result, a diameter of 5 mm and width of 2 mm were most appropriate. The device opened even when wet from gastric or intestinal juice. If getting wet with the gastric juice when exceeding 5 mm, the ring became unable to open fully. As to width, stored in a sheath, most operable size was 2 mm. As to thickness, sufficient expansion was obtained at 100 μm; however, since a 100 μm was rather difficult to operate when inserting another clip into the ring, a 200 μm excelled most in operability having a certain degree of stiffness, 300 μm could not obtain sufficient elongation rate. Therefore, we chose 200 μm. The maximum elongation rate against a load was 830 % against a load of 1,633 g. The required load was not proportional to the elongation rate. Our results showed that when a long elongation rate was required, the load needed to achieve such an elongation rate increased exponentially (Fig. 7). For grasping a lesion, the most suitable size of the hemoclip was 6 mm. With a shorter length, the force in grasping the lesion is weakened and with a longer length the hemoclip becomes obstructive in the case of release. As depicted in Fig. 8, the maximum size lesion in the pig’s stomach that is possible to dissect with use of one device was 70 mm. Theoretically, expansion of the device could only apply to lesions a maximum of 41.5 mm because of 5 mm × 830 %. However, because margins of the lesions were trimmed and the actual treated lesions were not planes, the device was possible to use under the endoscope for a lesion a maximum of 70 mm (Fig. 8).

### Study patients

Thirty-seven cases (device used group) were enrolled in this study, and the same number of patients with lesions matched for size and location were selected randomly from a database as control subjects (device not used group). Table 1 shows age, sex, and size of the resected specimen in both groups (Table 2).

**Table 1:** Study subjects

	Device not used (*n* = 37)	Device used (*n* = 37)
Median age (range)	70.2 (57–86)	70.4 (56–82)
Male/female	26/11	26/11
Mean size (range)	32.6 mm (14–76)	39.8 mm (21–90)

**Table 2:** Optimal configuration of the medical ring

Color	
Pink	Poor visibility
Green	Poor visibility
White	Good visibility
Black	Good visibility
Reviews of diameter (mm)	
5	Even if it gets wet, it opens and can be stored in sheath
10	Device will not open if it gets wet
15	Device will not open if it gets wet
Width (mm)	Operability
0.5	Poor
1	Poor
1.5	Good
2	Excellent
2.5	Good
3	Poor
Reviews of thickness (μm)	
20	Device will not open if it gets wet
40	Device will not open if it gets wet
60	Device will not open if it gets wet
80	Device will not open if it gets wet
100	Even if it gets wet, it opens; however, operability is affected to a certain degree
200	Good operability and sufficient growth was obtained
300	Sufficient growth was not obtained

### Comparison of dissection time of device not used and device used groups

Regarding subgroups of both the device not used and device used groups assigned according to treatment difficulty, 12, 19, and 6 cases belonged to group A (easy treatment), B (intermediate treatment), and C (difficult treatment), respectively. All lesions were resected en bloc, with free lateral and vertical margins. There was no significant bleeding that required blood transfusion or perforation related to the procedures. Comparison of average dissection time per cm^2^ of device not used group versus device used group was: group A, 5.8:2.1 min/cm^2^ (*p* < 0.01); group B, 6.1:3.8 min/cm^2^ (*p* < 0.01); group C, 7.9:3.6 min/cm^2^ (*p* < 0.01), respectively. Dissection time in the device groups was significantly shorter than in the device not used groups regardless of location of the lesion as shown in Table 3. No complication occurred in either group.

**Table 3:** Dissection time per cm^2^ of each group

	Device not used (min/cm^2^)	Device used (min/cm^2^)	Significant (*p*)
Overall	6.3 ± 3.6	3.18 ± 2.29	<0.01
	*n* = 37	*n* = 37	
Group A	5.8 ± 4.34	2.1 ± 1.54	<0.01
	*n* = 12	*n* = 12	
Group B	6.1 ± 3.44	3.8 ± 2.64	<0.05
	*n* = 19	*n* = 19	
Group C	7.9 ± 2.39	3.6 ± 1.72	<0.01
	*n* = 6	*n* = 6	

Group A, easy: anterior wall, posterior wall, greater curvature of antrum; Group B, intermediate: front view of antrum, lesion over an angle, lesser curvature of corpus; Group C, difficult: greater curvature of corpus, fornix. Data are mean ± SD unless otherwise indicated

## Discussion

Traction systems that facilitate ESD, such as PTA-EMR [8], the magnetic anchor system [9], external grasping forceps [10], peroral traction-assisted ESD [11], spring devices [12], clip-band technique [13], etc., have been described, although each has its own unique limitations. PTA-EMR, which requires a laparoscopic port with a trocar, is very invasive and expensive and necessitates use of an operating room and anesthesia [8]. The magnetic anchor system requires a large, expensive control device that is not yet available for clinical use [9]. A grasping forceps is inflexible at the time of dissection, due to its moving in line with movement of the scope and requires an assistant operator. In addition, grasping methods cannot be used for lesions in distal locations, because there is no space to maneuver the J-turn of scopes [10]. With peroral traction-assisted ESD lesions can be lifted only from the anal side, there are many restrictions due to the affected region, and, particularly, it cannot be used for sites where inversion operations cannot be performed [11]. The spring device needs a special hemoclip and sheath. When a spring device cannot be retrieved, when it is separated within the stomach and is performed through the intestine, there is the possibility of an intussusception that it will remain in the intestine or result in intestinal perforation. In addition, because the spring device is made of stainless steel, its safety within the intestinal tract has not been established. When a hemoclip on the body of the device is torn off when collecting the device, there is a risk of causing a postoperative hemorrhage, at least from the portion where the hemoclip was torn away [12]. The clip band technique is similar to the medical ring, but a bilateral approach cannot be used with that method; in addition, the report of its use is an animal study and it has not compared with the standard ESD technique [13]. There has been no report of the effects on the human body of other traction devices that are detached within the stomach and eliminated through the intestine.

Because the medical ring that we prepared is made from material used in approved medical devices, it can be sterilized and has been shown that if collection becomes impossible, it is not problematic. Through use of the medical ring assisted traction method described herein to facilitate ESD, direct visualization of the submucosal layer is obtained and an appropriate amount of tension by the elastic body on the submucosa can be applied to facilitate rapid dissection. Because the medical ring is only 5 mm in diameter, it maintains a good field of vision till resection is completed. This new device uses only surgical nylon and a conventional hemoclip, both commonly available. Because it can be easily attached on a hemoclip, no other tool is required.

Since this device can be connected under the endoscope to provide traction to both sides of the lesion, it is not restricted due to the site or the width of the lumen and, unlike with traction on only one side, it is possible to approach the lesion from both sides. That connection can be made with several portions of larger lesions or sites that are difficult to treat, which enables further improvement of the visual field. If connected with 3–0 silk, two pieces can be stored in a sheath, and it will be possible to connect them with clips on the outside of the sheath.

Although many traction devices reported thus far provide traction on only one site, with the present device traction can be applied to two or more places, there is no limit to the size of the excised lesion, and the device does not remain inside the body after the excision. The hemoclips are atraumatic to tissue and are removed with the resected specimen. In summary, the medical ring that we developed is simple, noninvasive, cost effective, and safe. Furthermore, exfoliation can be accomplished rapidly with tension by the elastic material, differing from devices that only improve the visual field.

There have been a few reports comparing groups using and not using devices to assist in ESD [9, 12], but these reports do not describe the location of the lesion or degree of difficulty in excising the lesion. The execution time in ESD has been examined by dividing locations and sizes in some reports [19–22], but locations just classified into three parts: upper, middle, and lower [upper portion (U), middle portion (M), and lower portion (L)]. However, when lesser curvatures and greater curvatures are compared even in the same portion, the degree of difficulty greatly differs, because there are higher levels of fat and thicker blood vessels in the greater curvatures of U and M. The degree of difficulty varies even in the same M depending on whether crossing over angles or not, and increases in L of lesser curvatures because the knife is perpendicularity applied. The degree of difficulty is significantly different depending on the location of the lesion; therefore, we have classified the difficulty level of treatments into three groups, considering not only the difficulty level of treatments in U, M, and L but also the difference of lesser curvatures and greater curvatures, the situation to crossover angles or not, and the operability of knives being poor or not. Moreover, as previously reported, it is quite natural that the larger the size, the larger the dissection area and the longer the operation time [19–22]. We considered that dissection time per cm^2^ does not be affected by the size and location. Therefore, to estimate the efficacy of this medical ring, comparison of dissection time per cm^2^ was important, not classifying it with sizes. Therefore, we examined various levels of difficulty in excision. Not only was the visual field vastly improved, but the exfoliation time was shorter with use of traction by the medical ring in all groups regardless of the degree of difficulty compared with groups matched for difficulty in which the device was not used. The treatment was performed with superior results by operators having experience from the elementary to the intermediate levels and significant differences were shown compared with groups with all degrees of treatment difficulty in which the device was not used. Securing a good cut line by traction enables the beginner to treat ESD rapidly and without complications. The possibility of the application of the device to the esophagus and large intestine is a future consideration.

## Conclusions

This newly developed traction device, the “medical ring,” which allows for good visibility, resulted in a shortened dissection time without any complications. Its use could facilitate the ESD procedure.

## Electronic supplementary material

Below is the link to the electronic supplementary material.

Video 1 Mounting method of medical ring. The most important point is to insert the device between the clip blades so that it will not be caught inside the sheath at the time of release (WMV 11958 kb)

Video 2 Medical ring assisted ESD (WMV 14341 kb)

Video 3 Bilateral approach to the lesion (WMV 12036 kb)

Video 4 Multiple connections to a large lesion (WMV 20394 kb)
